# Liquid Chromatographic Determination of Flavoxate HCl in Pharmaceutical Formulation

**DOI:** 10.4103/0975-1483.66787

**Published:** 2010

**Authors:** M Attimarad

**Affiliations:** Department of Pharmaceutical Sciences, College of Clinical Pharmacy, King Faisal University, Al-Ahsa, KSA

**Keywords:** Flavoxate HCl, high performance liquid chromatography, validation

## Abstract

The objective of the study was to develop a high performance liquid chromatography (HPLC) method using ultra violet (UV) detection for the determination of flavoxate HCl in bulk and solid dosage forms by using ibuprofen as the internal standard. Eclipse C18 column (150 mm × 4.6 mm, 5 μm) was used as the stationary phase with a mixture of acetonitrile : 0.1% formic acid in water (75: 25 v/v) as the mobile phase. The response of the drug was linear in the concentration range of 1 – 250 μg/ml. Limit of detection and Limit of quantification were found to be 0.23 μg/ml and 0.69 μg/ml, respectively. The percentage of recovery ranged between 97.4 and 101.3%. The factors affecting column separation of the analyte were studied. The results demonstrated that this method is reliable, reproducible, and suitable for routine quantitative use.

## INTRODUCTION

**Figure 1:**
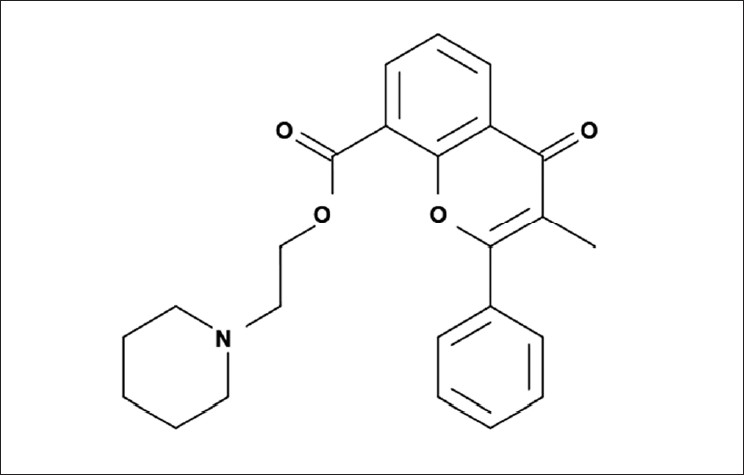
Structure of Flavoxate HCl

Flavoxate hydrochloride (FLX, 3-methylflavone-8-carboxylic acid β-piperidinoethyl ester hydrochloride, C_24_H_26_ ClNO_4_, MW : 427.93, Figure 1), belongs to a series of flavone derivatives, which exhibit strong smooth muscle relaxant activity, with selective action on the pelvic.[1] It is used for the symptomatic relief of pain, urinary frequency, and incontinence associated with inflammatory disorders of the urinary tract. It is also used for relief of vesicourethral spasms resulting from instrumentation or surgery.[2]

The literature survey reveals that FLX was estimated from its pharmaceutical preparations by using Ultraviolet spectrophotometry,[3,4] high performance liquid chromatography (HPLC),[5–9] Voltammetry,[10] capillary electrophoresis,[11] and potentiometric[12] determination techniques. The official method for the determination of FLX is non-aqueous titration using perchloric acid as a titrant, in pure form, and spectrophotometry in tablets.[4] Gilliland *et al*.[13] reported the LC-MS / MS determination of Flavoxate and its metabolite 3-Methylflavone-8-Carboxylic acid (MFA) in Human NaF Plasma.

The retention time of the reported HPLC methods is more than five minutes and the flow rate of the mobile phase is 1.5 ml/min, which requires a greater mobile phase for the determination of flavoxate. Hence, in the present study it was intend to develop a rapid, economical, simple, and precise RP HPLC method for the estimation of flavoxate HCl in bulk and formulations.

## EXPERIMENTAL

### Chromatographic conditions

The HPLC Agilent 1200 system (Germany) was equipped with an auto injector (100 μl syringe) and connected to a variable diode array wavelength detector and recorder. A Zorbax eclipse XBD-C18 reversed phased column (150 mm × 4.6 mm. i.d.) bonded on to 5 μm silica gel, manufactured by Agilent, was used for analysis. A mixture of acetonitrile : 0.1% formic acid in water (75 : 25 v/v, pH 3.0) was used as the mobile phase. The flow rate was 0.8 ml/min, wavelength was 218 nm, and the injection volume was 20 μl. The analysis was carried out at ambient temperature.

### Reagents

All the chemicals were of analytical grade and used without further purification. The water was distilled and deionized by passing it through the water purification system. HPLC grade Acetonitrile (Chromasolv) and AR grade formic acid from Sigma Aldrich (Switzerland) were used in this study. The standard drug samples of the flavoxate HCl and ibuprofen used were supplied by Mankind Pharma Ltd., India.

### Standard and sample preparation

Standard stock solution at a concentration of 1 mg/ml FLX was prepared by dissolving an appropriate amount of the standard drug in the mobile phase. Internal standard ibuprofen (IBU) solution was prepared by dissolving 10 mg of IBU in 10 ml of the mobile phase. Twenty tablets of FLX as HCl salt each containing 200 mg were weighed and powdered. A quantity equivalent to 10 mg of FLX, as HCl salt powder, was weighed and transferred to a 10 ml volumetric flask containing 8 ml of the mobile phase and sonicated for 10 minutes. Subsequently, the volume was made up to the mark with the mobile phase. The solution was filtered using a nylon 0.45 μm membrane filter. This solution was diluted with the mobile phase to get the concentration in the Beers range.

### Method validation

The method validation parameters, such as, specificity, linearity, accuracy, precision, limit of detection, limit of quantification, and robustness were verified as per the International Conference on Harmonization (ICH) guidelines.[14]

### Specificity

The specificity of the method was confirmed by comparison of chromatograms obtained from the standard and the samples.

### Linearity and range

Appropriate aliquots of standard stock solution were taken in 10 ml volumetric flasks. Internal standard IBU solution (0.5 ml) was added to all the volumetric flasks and the volume was brought up to the mark with the mobile phase to get the following concentrations: 1, 10, 50, 100, 150, 200, and 250 μg/ml of FLX and 50 μg/ml of IBU. Twenty microliters of the standard and sample were injected and the chromatograms were recorded.

### Accuracy (% Recovery)

The accuracy study was carried out by the standard addition method. Known amounts of standard solution of FLX (50, 100, and 150 μg/ml) were added to a sample solution of FLX (50 μg/ml), with IBU as the internal standard (50 μg/ml). Each solution was injected in triplicate and the percentage recovery was calculated by calculating the ratios of the peak areas.

### Precision

Standard solutions having concentrations of 1, 10, 50, 100, 150, 200, and 250 μg/ml of FLX and 50 μg/ml of IBU were prepared. For the intra-day studies, three repeated measurments of these solutions were carried out within a day and %RSD was calculated. In the inter-day variation studies, three repeated measurements of the same solutions were carried out on three consecutive days and %RSD was calculated.

### Limit of detection and limit of quantification

The limit of detection (LOD) and limit of quantification (LOQ) were calculated from the standard deviation of responses and slopes using signal-to-noise ratio as per ICH guidelines.[14]

### Robustness

Robustness of the method was studied by changing the extraction time of FLX, from dosage form by five minutes, composition of mobile phase by 2% of organic phase, flow rate by 0.1 ml/min, and wavelength by 1 nm.

### System suitability test

System suitability tests were used to verify the resolution and repeatability of the system. The parameters used in this test were asymmetry of the chromatographic peak, peak resolution, and repeatability, as *relative standard deviation* (RSD) of the peak area, for repeated injections. The precision of the instrument was checked by repeatedly injecting a solution of FLX (100 μg/ml).

## RESULTS AND DISCUSSION

**Figure 2:**
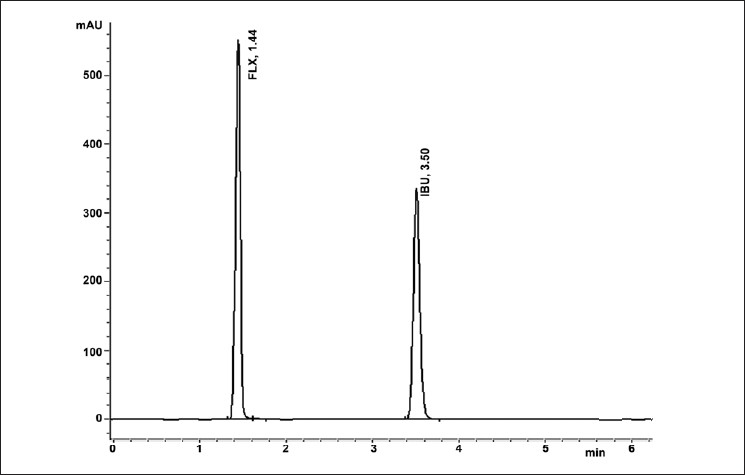
Representative chromatograms of Flavoxate HCl (50 μg/ ml) (Rt 1.44) and internal standard Ibuprofen (50 μg.mL-1) (Rt 3.50) measured at 218 nm

Chromatographic conditions were developed empirically by selecting various solvents such as water, acetonitrile, and methanol in different concentrations, and the retention time and resolution were assessed to get optimum conditions. Under the established chromatographic condition, flavoxate HCl and internal standard (IS) peaks were well-resolved. As the system suitability parameters such as tailing factor and theoretical plate numbers were within the acceptable limits, the resolution of peaks were considered optimum. Retention time for FLX was 1.44 minutes, whereas, for IBU it was 3.50 minutes [Figure 2], indicating the rapid analysis of FLX. The calibration curves were constructed by plotting ratios of the peak areas of FLX and peak areas of IBU versus concentration of FLX, and the regression equations were calculated. Each response was the average of three determinations. The results [Table 1] obtained showed that the linearity range was 1 – 250 μg/ml. The corresponding regression equation was found to be: Y = 0.022 (X) + 0.0052 (Y is peak area ratio of FLX to IS and X is FLX concentration) with a correlation coefficient of 0.9995.

**Table 1:** Summary of regression analysis and validation parameters

Parameters	Values*
Regression analysis	
Slope	0.022
Intercept	0.0052
Correlation coefficient	0.9995
Validation parameters	
LOD (μg/ml)	0.23
LOQ (μg/ml)	0.69
Accuracy (%) ± % RSD	99.6 ± 1.51
Precision (% RSD)	
Intra-day (n = 3)	0.05 – 0.43
Inter-day (n = 3)	0.15 – 0.65
Repeatability (% RSD)	0.84
System suitability test parameters	
Retention time (min) ± % RSD	1.44 ±0.05
Tailing factor ± % RSD	1.03 ± 0.1
Theoretical plates ± % RSD	11169 ± 1.95

The recovery study showed that the mean recovery was 99.6% and the RSD for three samples was lower than 2.0%. The RSD values of intra-day and inter-day were found to be in the range of 0.05 – 0.43 and 0.15 – 0.65, respectivly. As these parameters were within the acceptable range, it was inferred that the method was precise. The accuracy of the developed method was assessed by the standard addition method and the RSD was found to be within the acceptable limit [Table 2]. The limit of quantification (LOQ) of the method, defined as the minimum concentartion that could be measured, was found to be 0. 69 μg/ml. The limit of detection (LOD) was 0.23 μg/ml [Table 1]. The method was found to be robust as the results were not significantly affected by the slight variation in the extraction time, composition of mobile phase, flow rate, and wavelength. The chromatographic parameters such as selectivity and peak symmetry were within the range, and %RSD of repeatability was found to be 0.84, which was found to be satisfactory. The number of theoretical plates and the tailing factor were observed to be satisfactory [Table 1].

**Table 2:** Recovery study data of FLX

Std. FLX conc. mcg/ml	Sample FLX conc. mcg/ml	Total amount of FLX from Std. graph mcg/ml*	Recovery of standard drug mcg/ml	% Recovery of standard
50	50	98.7	48.7	97.4
100	50	151.3	101.3	101.3
150	50	197.6	147.6	98.4

* Mean value of the three determinations

### Analysis of marketed formulation

The proposed validated RP-HPLC method was successfully applied to determine FLX in two marketed formulations. The mean percentages of FLX were found to be 99.6 ± 1.51 and 101.1±1.02, which were comparable to the corresponding labeled amounts [Table 3]. Furthermore, the matrix components, for example, the excipients, did not interfere with the analyte.

**Table 3:** Assay result for Flavoxate HCl (200 mg per tablet) in the formulation product

Formulation No.	FLX in label claim (mg)	Total amount of FLX found (mg)*	% of FLX found	% RSD
1	200	199.2	99.6	1.5
2	200	201.6	101.1	1.0

* Mean value of the three determinations

## CONCLUSION

The validated RP-HPLC method employed here has proved to be rapid, simple, specific, accurate, precise, sensitive, and robust. It can be successfully used for the routine analysis of FLX in bulk and in solid dosage form.
